# Quality Changes during Frozen Storage of Mechanical-Separated Flesh Obtained from an Underutilized Crustacean

**DOI:** 10.3390/foods9101485

**Published:** 2020-10-17

**Authors:** Silvia Tappi, Ana Cristina De Aguiar Saldanha Pinheiro, Dario Mercatante, Gianfranco Picone, Francesca Soglia, Maria Teresa Rodriguez-Estrada, Massimiliano Petracci, Francesco Capozzi, Pietro Rocculi

**Affiliations:** 1 Department of Agricultural and Food Sciences, Alma Mater Studiorum—University of Bologna, 47521 Cesena, Italy; anacristin.deaguiar2@unibo.it (A.C.D.A.S.P.); gianfranco.picone@unibo.it (G.P.); francesca.soglia2@unibo.it (F.S.); m.petracci@unibo.it (M.P.); francesco.capozzi@unibo.it (F.C.); pietro.rocculi3@unibo.it (P.R.); 2 Interdepartmental Centre for Agri-Food Industrial Research, Alma Mater Studiorum—University of Bologna, (FC) 47521 Cesena, Italy; maria.rodriguez@unibo.it; 3 Department of Agricultural and Food Sciences, Alma Mater Studiorum—University of Bologna, 40127 Bologna, Italy; dario.mercatante2@unibo.it

**Keywords:** *Squilla mantis*, mantis shrimp, mechanically separated flesh, frozen storage, lipolysis, lipid oxidation, proteolysis, quality changes

## Abstract

Despite their high nutritional value, high quantities of fish caught in the Adriatic Sea are underused or discarded for their insignificant economic value. Mechanical separation of flesh represents an opportunity for developing innovative semi-finished products, even if it can promote an increased quality degradation rate. The aim of this study was to evaluate physico-chemical modifications of mechanically separated mantis shrimp flesh during deep-freezing storage. Flesh samples obtained using a belt-drum separator, frozen and vacuum-packed, were stored at 3 temperatures (industrial: −26 °C; domestic: −18 °C and abuse: −10 °C) for 12 months. During storage, qualitative (color, water content, pH, fatty acids (FA) and lipid oxidation) were evaluated. Fish freshness parameters (e.g., trimethylamine (TMA), dimethylamine (DMA) and amino acids) were assessed using nuclear magnetic resonance (^1^H-NMR). The mechanical separation process accelerated the initial oxidation phenomena, promoting color alterations, compared to manual separation. The main degradation phenomena during storage were significantly affected by temperature and were related to changes in luminosity, oxidation of n-3 polyunsaturated fatty acids (PUFA), increased lipolysis with release of free FA, production of TMA and DMA by residual enzymatic activity, and changes in amino acids due to proteolysis. The inter-disciplinary approach permitted important findings to be made, in terms of the extent of different degradative phenomena, bound to processing and storage conditions of mechanically separated mantis flesh.

## 1. Introduction

Despite its high nutritional value, a high quantity of fish caught in the Adriatic Sea is underused or discarded mainly due to its insignificant local economic value or for regulatory reasons [1]. In the Adriatic Sea, bottom trawling represents 40% of total landings and the mean discard rate of this fishing gear ranges between 20% and 67% of total catches [2]. Moreover, many seafood products are characterized by significant changes in quality and abundance throughout the year. Therefore, it would be convenient, from both economic and sustainability standpoints, to promote strategies for their valorization, which lead to the development of innovative, high-added value products with prolonged shelf-life and that are available throughout the year, thereby reducing waste.

Mechanical separation is a technology that is successfully applied in the fish sector, even though the loss or modification of the normal structure of the muscle fiber often occurs during this operation [3]. Mechanical separation of fish flesh could represent an opportunity for the development of fish-based innovative products from seafood that would otherwise be discarded.

Moreover, the by-products of mechanical separation of crustaceans (shells and carapaces) could be further exploited for the extraction of valuable compounds (such as chitin and chitosan) that have several uses in the food industry (i.e., anti-microbial, antioxidant, and anti-inflammatory agents), as well as in the non-food sector [4], contributing to increase the sustainability and the economic value of the overall food chain.

However, this type of preparation process could lead to an increased quality degradation rate of an already highly perishable product. Indeed, besides microbial proliferation, fish are also highly susceptible to lipid and protein oxidation, which can promote the production of biogenic amines and other compounds considered hazardous compounds [5]. Storage under deep-freezing conditions greatly increases the shelf-life of seafood products by arresting microbial growth and, in general, slowing down all other chemical and enzymatic degradation reactions. Freezing could help provide high-quality product constantly throughout the year and prevent product waste. However, freezing temperatures are only able to slow down enzymatic activity and oxidation. The main limiting factor of the shelf-life of frozen fish products is, in fact, represented by lipid degradation, due to both oxidative and hydrolytic reactions which affect their nutritional and sensory profile.

From an economical point of view, the whole process mainly requires a mechanical deboner and a temperature blast chiller. While many companies working in the seafood sector generally possess a blast chiller, the mechanical deboner, often used in the poultry sector, may represent the main initial cost of investment. However, it can be considered relatively inexpensive processing equipment that does not require big changes in production lines and that is also characterized by limited energy consumption, confirming its suitability to valorize underutilized or low-value species.

The mantis shrimp (*S. mantis*) is a common specie along the coast of the Mediterranean Sea and it is one of the most important resources in the northern and central Adriatic Sea, due to its easy capture and commercial/economic value, where it represents 66% of the demersal fisheries mainly caught by bottom trawlers [6]. Among the crustaceans it is characterized by a low market value, nevertheless, is can be considered a good source of n-3 and n-6 polyunsaturated fatty acids (PUFAs). Moreover, the high levels of essential amino acids can make it an alternative source of proteins for the populations of developing countries [7]. However, it is characterized by a marked seasonality, with the highest values occurring in winter and the lowest in April–July [8] and a large discarded amount due to its small size.

The aim of this study was to evaluate the modifications of some qualitative indices of mechanically separated mantis flesh during deep-freezing storage. The mantis shrimp was separated, deep-frozen, vacuum-packed and stored at three different temperatures that represented domestic storage (−18 °C), industrial preservation (−26 °C) and thermal abuse condition (−10 °C). Different qualitative indices (pH, dry substance, color, main lipid classes, fatty acid (FA) composition, thiobarbituric acid reactive substances (TBARs), trimethylamine-N (TMA-N), trimethylamine-O (TMA-O), dimethylamine (DMA), lysine, alanine and sarcosine) were determined during storage.

## 2. Materials and Methods

### 2.1. Chemicals

All chemicals and solvents were of analytical grade. Methanol and *n*-hexane were purchased from Merck (Darmstadt, Germany); anhydrous sodium sulfate was supplied by BDH (Poole, England) The standard mixture of fatty acid methyl esters (GLC 463) was purchased from Nu-Chek (Elysian, MN, USA), whereas the tridecanoic acid methyl ester was supplied by Steraloids (Newport, RI, USA). All the other chemicals and standards were purchased from Sigma-Aldrich Chemical Company (St. Louis, MO, USA).

### 2.2. Raw Materials

Mantis shrimps (*S. mantis*) were fished in February 2017 in the Adriatic Sea (Food and Agriculture Organization (FAO) Major Fishing Area 37, Subarea 37.2.1) and subsequently kept in ice for 24 h before processing. Mantis shrimp flesh was obtained by either manual separation (FF, control group) or using a belt-drum separator (MSF). In the mechanical deboner model 600 (Baader, Germany), flesh was forced by means of a rubber conveyor belt through a perforated drum (holes diameter 3 mm) and collected from the inside of the drum, while carapaces were discarded on the outside. Flesh was divided into polypropylene (PP) trays of about 100 g each. 57 trays were frozen in a cooling system until reaching −26 °C at the sample core. Temperature was monitored by inserting thermocouples at the core of 4 trays. Once the samples were already frozen, the trays were vacuum-packed in a high barrier PP film.

### 2.3. Storage

The frozen and packaged samples were divided into three freezers (18 trays each) at temperatures of −26 °C, −18 °C and −10 ± 0.5 °C to simulate industrial, domestic and thermal abuse conditions, respectively. During storage, three trays per each temperature were sampled at different time intervals (0, 1, 2, 4, 6, 9 and 12 months), thawed at 4 °C for 16 h and analyzed.

### 2.4. Analytical Determinations

#### 2.4.1. Physico-Chemical Parameters

pH was measured by a pH meter (Crison, Barcelona, Spain). For each sampling time, the measurement was performed in triplicate. Water content was evaluated with a gravimetric method, measuring the weight difference before and after drying, until constant weight was reached in an oven at 70 °C. The measurement was performed in triplicate on each sample at each storage time. Color was evaluated with a portable tristimulus spectrum-photocolorimeter (Hunterlab ColorFlex™, Reston, VA, USA) using the CIELab scale with L*, a* and b* as color parameters. In the present study, the L* value (brightness indicator, with values between 0 and 100) and the red index a*, were considered. The color was measured in triplicate.

#### 2.4.2. Lipid Oxidation

Thiobarbituric acid reactive substances (TBARs) were determined according to Bao and Ertbjerg [9] and used as a lipid oxidation indicator. Samples (5 g) were homogenized in ice using a IKA Ultra-Turrax T25 homogenizer (Labortechnik, Staufen, Germany) at 13,000 rpm for 30 sec in 15 mL of 5% trichloroacetic acid (TCA) (w/v) and 0.5 mL of butyl hydroxytoluene (BHT) (4.2% w/v in ethanol). After filtering the homogenate through filter paper (Whatman 42), a 2 mL aliquot was taken and added with 2 mL of thiobarbituric acid (0.02 M). Thereafter, after incubating the solution at 100 °C for 40 min and cooling down the samples in an ice bath, the absorbance was measured at 532 nm with an ultraviolet (UV)-visible spectrophotometer (mod. UV-1800; Shimadzu, Kyoto, Japan). To calculate the amount of malondialdehyde (MDA) produced, a standard 1,1,3,3-tetraethoxypropane curve was used in the concentration range of 0.1 to 2.0 mM. Finally, TBARs content was expressed in mg MDA/kg sample. The measurement was performed in triplicate for each storage interval and for each temperature.

#### 2.4.3. Lipid Extraction

Lipid fraction was extracted from 50 g of samples using chloroform and methanol according to the modified Bligh and Dyer method [10]. The lipid content was determined gravimetrically, and the results were expressed as g lipid/100 g of the sample. The extraction was performed twice on each sample. Extracted lipids were stored at −40 °C until analyzed.

#### 2.4.4. Total Lipid Profile

The determination of the main lipid classes (free fatty acids (FFA), monoacylglycerols (MAG), free sterols (STE), diacylglycerols (DAG), esterified sterols (EST) and triacylglycerols (TAG)) were determined by gas chromatography-flame ionization detection (GC-FID) [9]. About 20 mg of lipid matter to which were added 0.251 mg of 5α-cholestane (internal standard) were dissolved in 1 mL of n-hexane: One μL of the solution was injected into a GC-FID (Shimadzu GC 2010 PLUS, Kyoto, Japan) under the same analytical conditions as Gallina Toschi et al. [11] (2014) and Luise et al. [12]. A fused silica capillary column (SE-52 MEGA, 10 m × 0.25 mm i.d. × 0.1 μm film thickness; Legnano, MI, Italy), coated with 95% dimethyl and 5% diphenyl polysiloxane, was used. The temperature was programmed from 100 to 355 °C at a rate of 5 °C/min and the final temperature was kept for 20 min. The injector and FID temperatures were set at 355 °C. Helium was used as carrier gas at a flow of 2.02 mL/min and a split ratio of 1:25. The different lipid classes were identified using diverse commercial standards (Sigma-Aldrich Chemical Company, St. Louis, MO, USA). The amount of each lipid class was determined using the internal standard method with the response factor of each main lipid class (estimated using suitable commercial standards), as reported by Luise et al. [12]. Two independent replicates were analyzed.

#### 2.4.5. Total Fatty Acid Profile

About 20 mg of lipid extract were methylated with 200 μL of diazomethane, added with 0.6 mg of tridecanoic acid methyl ester (C13, internal standard), transmethylated with 40 μL of 2 N potassium hydroxide (KOH) in methanol [13], vortexed for 1 min, left standing for 5 min, and centrifuged at 1620× *g* for 5 min. One microliter of supernatant was injected into a GC-FID (GC8000 series, Fisons Instruments, Milan, Italy), interfaced with a data acquisition system (Chromcard Data System, ver. 2.3.1, Fisons Instruments). A Restek RTX 2330 fused-silica column (30 m × 0.25 mm × 0.2 μm film thickness) (Bellefonte, PA, USA) coated with 90% biscyanopropyl and 10% cyanopropyl-phenyl polysiloxane, was used. Oven temperature was programmed from 100 °C to 240 °C at 5 °C/min, and kept at 240 °C for 20 min. Both injector and detector temperatures were set at 250 °C. Helium was used as carrier gas at a constant pressure of 75 KPa and a split ratio of 1:30. Peak identification was performed by comparing the retention times with those of the GLC 463 FAME standard mixture. Tridecanoic acid methyl ester was used as internal standard for FA quantification; the GC response factor of each FA was calculated by using the GLC 463 FAME standard mixture and the internal standard. Limit of detection (LOD) and limit of quantification (LOQ) were calculated as signal-to-noise ratios equal to 3:1 and 10:1, respectively.

FAME quantification was performed according to the following formula:

Qi = (Ai × Qis)/(Ais × W × Kris)

where Qi is the FA concentration (mg/100 mg), Ai is the FA peak area, Qis is the concentration of the internal standard (C13 methyl ester, mg), Ais is the internal standard peak area, W is the weight of the lipid sample (mg) and Kris is the response factor.

#### 2.4.6. Nuclear Magnetic Resonance (^1^HR-NMR) Metabolomics for Quality Indexes

Samples were prepared as reported by Picone et al. [14]. Briefly, for each sample, an extraction with 7% of perchloric acid solution was performed in triplicate. The acid mixtures were neutralized to pH 7.8 using 9 M KOH and then centrifuged at 14,000 rpm for 10 min at 4 °C in order to remove potassium perchlorate precipitate. We added to 720 μL of supernatant 80 μL of 3-(trimethylsilyl)-propionic-2,2,3,3-d4 acid sodium salt (TSP) 10 mM and then centrifuged it one more time at 14,000 rpm for 10 min at 4 °C. Samples were transferred to a 5 mm NMR tube and spectra were acquired and processed using parameters reported in previous researches [15,16].

Signals were assigned by using a multimedia library included in Chenomx NMR Suite 8.2 professional software (Chenomx, Edmonton, AB, Canada) and the concentration of trimethylamine-N (TMA-N), trimethylamine-O (TMA-O), dimethylamine (DMA), lysine, alanine and sarcosine were determined as indicators of fish freshness.

### 2.5. Statistical Analysis

Differences between mean values of manually and mechanically separated flesh were analyzed by a *t*-test (*p* < 0.05). Storage data were analyzed using two-way analysis of variance (ANOVA) including storage time (St) and temperature (T) and their interaction (St T) as factors. Means were separated by Tukey’s honest significance test (*p* < 0.05). Pearson’s analysis (*p*-level < 0.05) was performed to evaluate the correlation between data. Principal component analysis (PCA) was used as explorative technique to discriminate the samples and to display the correlation between the parameters. Statistical analysis of the data was performed by using the software Statistica 8.0 (StatSoft Inc., Tulsa, OK, USA)

## 3. Results and Discussion

### 3.1. Comparison between Manually and Mechanically Separated Fresh Flesh

In Table 1, the physico-chemical parameters and fatty acid (FA) composition of manually and mechanically separated flesh, are compared. No significant differences were observed for water content and pH. On the contrary, the color parameter L* showed a significant variation; indeed, after mechanical separation, the flesh appeared darker. Secci et al. [17] detected a similar color variation in horse mackerel after mechanical separation. The initial TBARs value was similar to that found by Sundararajan et al. [18] in frozen shrimp. However, a significant increase of this parameter was observed, indicating that the mechanical separation process promoted lipid oxidation; similar results were detected by Secci et al. [3] in two different fish species.

The fat content of the flesh varied from 2.8% to 3%. This value is in agreement with literature results [7,8,9,10,11,12,13,14,15,16,17,18,19] and did not change according to the different separation method.

Regarding lipid classes, no significant differences were observed between the manually and mechanically separated fresh flesh. It must be noted that DAG, EST and TAG were the most abundant lipid classes in both types of mantis flesh, evidencing already a hydrolytic process of lipids. Concerning the total FA composition (Supplementary material—Table S1), it remained practically unchanged in both manually and mechanically separated fresh flesh, even though mechanical separation led to higher oxidation which was reflected in a significant decrease of some unsaturated FA (C16:1 n-7 and C20:3); similar results were obtained by Secci et al. [17] on horse mackerel flesh. The main FA were oleic acid (C18:1 n-9, 16.9 ± 0.2% of total FA) and palmitic acid (C16:0, 16.4 ± 0.5% of total FA), followed by palmitoleic acid (C16:1 n-7, 14.6 ± 0.4% of total FA) and nervonic acid (C24:1 n-9, 14.6 ± 0.5% of total FA). Eicosapentaenoic (C20:5 n-3, EPA) and docosahexaenoic (C22:6 n-3, DHA) acids were present as 5.26 ± 0.36% and 0.88 ± 0.02% of total FA, respectively. Among FA categories, monounsaturated FA (MUFA) were the more abundant, followed by saturated FA (SFA) and PUFA. In particular, SFA represented about 30% of total lipids and were mainly composed by C16:0 and C18:0; in the case of MUFA, C18:1 n-9 and C16:1 n-7 represented about 60% of this FA class. On the other hand, PUFA accounted for 13% of total lipids and, while more than 90% was constituted by PUFA n-3 (in particular EPA and DHA), only 2.8% was represented by PUFA n-6. These values are within the ranges of FA percentage distribution reported by Mili et al. [7] for *S. mantis* fished in Tunisian waters in different seasons, as well as those found by Passi et al. [19] for Mediterranean mantis shrimp. In fact, it is well known that FA composition of fish lipids can be affected by species, genetic, physiological, morphological, dietary, seasonal and environmental factors, among others [20,21]. The PUFA n-6/PUFA n-3 ratio, suitable index to compare the nutritional value of food, was around 3.6 and no significant differences were observed with respect to the separation process used. According to Simopoulos [22], a low PUFA n-6/PUFA n-3 ratio (< 4) is desirable for a healthy human diet. This result confirms the importance of Mediterranean mantis shrimp as a rich dietary source of PUFA n-3.

### 3.2. Variation of Quality Indices during Frozen Storage

During the storage at three different temperatures, pH varied from 6.49 to 6.71, while moisture content ranged from 85.59% to 86.62% (pH and moisture data not reported); in both cases, no significant differences were observed across storage at the different temperature conditions.

**Figure 1:**
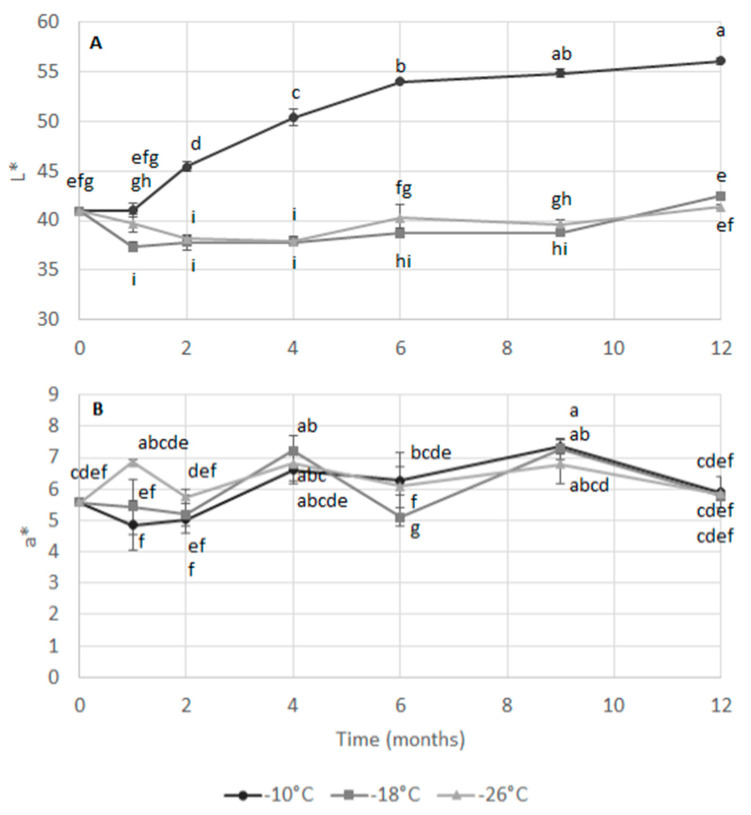
Colorimetric parameters of luminosity (L*) (**A**) and red index (a*) (**B**) of mechanically separated mantis shrimp flesh during frozen storage at −10, −18 and −26 °C. Different letters indicate significant differences (at *p* < 0.05) among samples.

Figure 1 shows the evolution of the colorimetric parameters of luminosity L* (Figure 1A) and red index a* (Figure 1B) measured in the mechanically separated *S. mantis* flesh during frozen storage at the three selected temperatures.

Evolution of color during storage can be associated with structural changes [23], as well as variations in pigments concentrations and their oxidative status [24]. While at the temperatures of −18 °C and −26 °C the L* values were roughly constant (37–42) throughout the 12-months storage, the sample stored at −10 °C showed a significant increase during the entire storage period, reaching values of 56. This parameter was significantly influenced by storage temperature, time and their interaction (Table 2). By contrast, the red index was significantly affected only by storage time and by the interaction between time and temperature. Although some significant variations were observed among samples during storage, there was not a clear trend and values remained between 5 and 7.

Sundararajan et al. [18] observed an increase in a* value for peeled frozen shrimp stored at –21 °C for 180 days, while no significant changes in L* values were observed. These authors suggested that the decrease in a* values could be mainly attributed to the degradation of astaxanthin and lipid oxidation.

To the best of our knowledge, there are no previous reports about the storage of mechanically separated flesh obtained from crustaceans, hence it is impossible to directly compare our results. Changes in the mechanically separated fish flesh obtained from horse mackerel [16], as well as from gilted sea bream, sea bass and rainbow trout [3], were evaluated during frozen storage, showing that color variations depended on the species considered. Shrimp flesh is highly perishable and normally high product quality can be obtained when immediately frozen after capture [25]. Generally, results showed that the main color differences occurred during processing rather than during storage and that white flesh led to lower changes, proving to be more suited for the development of fish processed products [3]. However, color fading, lipid oxidation, protein denaturation, and dehydration can occur during the frozen storage of shrimp and other crustaceans [25]. Color variations observed in the sample stored at −10 °C may be related to enzymatic and non-enzymatic reactions that result in degradation of myofibrillar proteins and disorganization of myofibrils. Chéret et al. [23] and Torres et al. [26] observed a similar change upon high hydrostatic pressure processing of sea bass fillets and horse mackerel, respectively.

**Figure 2:**
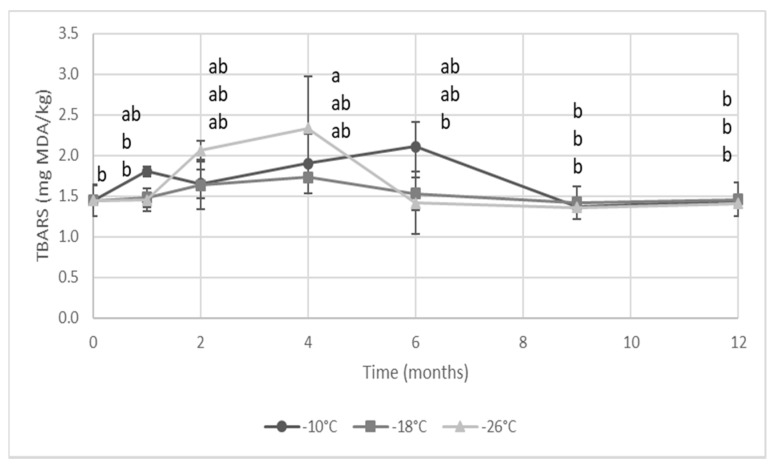
Thiobarbituric acid reactive substances (TBAR) values of mechanically separated mantis shrimp flesh during frozen storage at −10, −18 and −26 °C. Different letters indicate significant differences (at *p* < 0.05) among samples.

Figure 2 reports the TBARs values found in the *S. mantis* flesh during the frozen storage, which varied from 1.4 to 2.4 mg MDA/kg for all the considered period, without significant variations in all storage conditions. Despite the secondary lipid oxidation initially induced by the mechanical separation process, TBARs did not show a steady increase during storage as expected, being thus in disagreement with data reported by various authors for oxidative stability of crustacean flesh and minced fish during frozen storage [3,27,28,29,30]. Sundararajan et al. [18] found a value of 0.47 mg MDA/kg in shrimp that increased progressively during frozen storage up to 2.96 after 180 days. Tsironi et al. [25] observed an increased rate of TBARs formation with increasing storage temperature in frozen shrimp. However, in the present research, after the initial increase of TBARs during processing, no further oxidation was detected by means of this index.

Besides the data dispersion observed, other factors could have also contributed too, such as the type of packaging, the presence and amount of lipophilic (such as vitamin E) and enzymatic (i.e., GSH) antioxidants in mantis shrimp [19]. On the other hand, aldehydes deriving from lipid oxidation could have also interacted with other matrix components (such as proteins, amines and peptides) [30], thus leading to the formation of compounds (i.e., Schiff bases) that cannot be determined as TBARs. In fact, lipid and protein oxidations can occur independently or in parallel, but they can also interact with each other [30].

Table 3 reports the distribution of the main lipid classes (expressed as % of total lipids) and the main total FA classes (expressed as % of total FA) in mechanically separated *S. mantis* flesh, as related to storage conditions. The total fat content (% on flesh) was significantly affected only by storage time (St); however, no significant differences were observed among all the determined values.

Concerning the distribution of the main lipid classes, FFA was found to be influenced by both storage temperature and time, increasing from 9% up to around 40% in samples stored at −10 °C after 6 months. Similarly, MAG rose by increasing storage time and storage temperature, while TAG and DAG content showed the opposite trend. These results evidence the occurrence of lipid hydrolysis during frozen storage, being more intense at storage temperatures above −26 °C. The accumulation of FFA in frozen marine species is related to some extent with lack of acceptability. FFA, in fact, are known to cause deterioration of seafood products through their interaction with proteins and have been reported to exert a great effect on lipid oxidation development [26]. FFA have also been shown to oxidize faster than higher molecular-weight lipids, i.e., TAG and phospholipids, due to their higher accessibility caused by their lower steric hindrance to oxygen and other prooxidant molecules [27].

Regarding the total FA composition (expressed as % of total FA), significant differences were observed in all FA classes as related to both storage temperature and the interaction between time and temperature (St T), except for PUFA n-6 and the n-6/n-3 ratio. Among the single FA (Table S2), docosapentaenoic acid (DPA) was noticeably affected by the storage temperature. On the other hand, storage time did not show any significant effect on the main FA classes, but some single FA (such as linolenic acid) varied to a relevant extent depending on the time of storage. However, it must be noted that there was not a clear trend of the concentration of most single FA with respect to the temperature and time of storage (Table S2), which could depend on a dynamic equilibrium between their accumulation and conversion into other compounds (i.e., oxidized fatty acids, volatile compounds).

In general, the impact of the storage method and duration on FA content varies according to the fish species and seems to greatly depend on their total lipid content. In fact, Rudy et al. [31] observed that the effect of storage conditions was greatest in fish species whose lipid content was around 10–19%, while species with lower lipid content (< 10%), like *S. mantis* (2.9–3.1%) in the present study, are usually less affected. The lipid content relates to the taxonomic classification, environment (freshwater or marine), season and/or geographic location (warm or cool waters), and lipid storage; all these factors influence the FA content of fish tissue and their susceptibility to degradation under diverse storage conditions. Although long-chain PUFA are usually more prone to oxidation, Rudy et al. [31] observed that FA degradation was more a function of fish species rather than FA type, suggesting species-specific FA dynamics during storage, probably related to the total lipid content in the fish species. In our work, as well as in that of Rudy et al. [31], no specific FA or FA class consistently and preferentially underwent a change in quantity with increasingly poor handling and storage conditions, even though a decrease in the amount of some FA was observed over time. Besides the influence of lipid content on FA alterations in marine species, they may also depend on other factors like size, sex, diet, season, state when captured, microbial load, genuineness, presence of natural antioxidants, tissue type, number of lipases and their location in cells [31].

**Figure 3:**
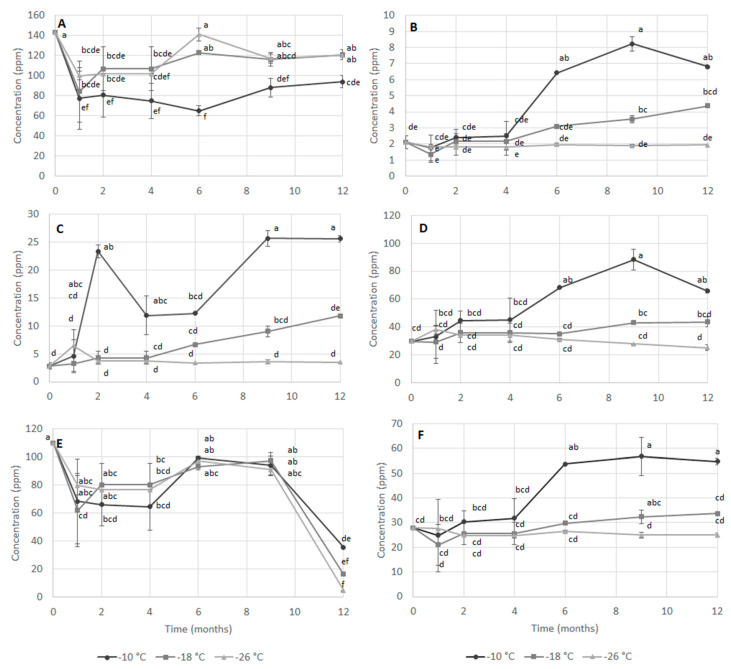
Concentration (ppm) of trimethylamine-O (TMA-O) (**A**), trimethylamine (TMA) (**B**), dimethylamine (DMA) (**C**), lysine (**D**), sarcosine (**E**) and alanine (**F**) measured by ^1^HR-NMR in extracts of mantis shrimp mechanically separated flesh during frozen storage at −10, −18 and −26 °C. Different letters indicate significant differences (at *p* < 0.05) among samples.

Figure 3 shows the concentration of some selected compounds present in the *S. mantis* flesh during storage and analyzed by ^1^HR-NMR. In Table 4, results of multivariate analysis show that all parameters were significantly influenced by storage time, temperature, and their interaction. TMA-O breakdown can occur via bacterial enzymes that release TMA [32], or by the activity of trimethylamine oxide demethylase (TMAOase) that leads to the formation of DMA and formaldehyde [33]. The production of TMA during refrigerated storage is considered an index of fish freshness as it is strongly correlated with microbial spoilage and it is characterized by a pungent, often associated with the typical “fishy” smell of seafood undergoing spoilage [34]. During frozen storage, bacterial activity should be absent; however, as mentioned earlier, TMA has also been reported to be a product of enzymatic degradation of TMA-O.

In the present study, TMA-O (Figure 3A) decreased rapidly in the first month and then remained fairly constant in samples stored at −18 °C and −26 °C. In the sample stored at −10 °C, instead, lower values were observed during the rest of the storage; in fact, at the end of the 12-month storage period, TMAO was half as much the initial value. In parallel to the decrease of TMAO, both TMA (Figure 3B) and DMA (Figure 3C) increased in samples stored at −18 and −10 °C, while roughly the same values were observed in the sample at −26 °C. In particular, TMA increased from the 4th month in both samples stored at −10 °C and −18 °C proportionally to the storage temperature. DMA started to increase after the first month for samples stored at –10 °C, whereas in samples kept at −18° C it rose just after 4 months and to a lower extent.

Sotelo et al. [35] found an increase of TMA during storage at −5 °C, but not at −12 °C. These authors suggested that some residual bacterial activity could still be found at temperatures slightly below zero. However, the TMA increase observed at −10 °C and −18 °C in the present study is probably related to enzymatic degradation. According to García-Soto et al. [27], the formation of TMA during frozen storage of crustaceans can also be due to biochemical breakdown of proteins and non-protein nitrogen (NPN) compounds.

Free amino acids in fish are the main components of non-protein nitrogen and, since some of them are precursors of aromatic components, they are directly responsible for the development of flavor and taste during cooking [36]. Amino acids have also been used as quality indices for various fish and crustacean species [37]. Some of them are precursors of biogenic amines obtained by decarboxylation, which are very important from the toxicity standpoint, and as quality control indices for fish spoilage.

During storage, changes in amino acids are caused by muscle autolysis and the concentration of the single components depends on a dynamic balance between their production and destruction, this balance being associated with muscle enzymes [37].

In the present study, starting from the 4th month, lysine (Figure 3D) and alanine (Figure 3F) began to increase more rapidly in sample at −10 °C and to a lesser extent in samples stored at −18 °C. In samples stored at –26 °C, by contrast, it remained constant. These results indicate a high level of proteolysis at storage temperatures above −26 °C. On the contrary, sarcosine (Figure 3E) displayed a variable trend. After a rapid decrease in the first months, it started to increase slowly in all samples until the 8th month and, thereafter, it decreased notably in all three samples.

### 3.3. Data Correlation

The Pearson correlation matrix of all data obtained during frozen storage of samples is reported in Table 5. Luminosity showed high positive or negative correlation with the majority of the other tested parameters, proving to be a valid indirect parameter for the quality determination of frozen flesh. By contrast, the red index (a*) was not correlated to any other parameters.

The use of TBARs for the determination of the oxidation level during storage only showed a significant correlation with TMA-O, confirming its low suitability for the discrimination of samples as described above.

The concentration of TMA-O, TMA and DMA were highly correlated to the content of lysine and alanine indicating that these components are strictly related to the protein breakdown occurring during shelf-life. They showed also highly significant correlation to some lipid classes, in particular FFA, MAG and TAG. This may indicate that the variation of all these indexes during storage at –10 °C is related to the same cause, probably a residual enzymatic activity. The relative content of the different FA classes instead did not show significant correlation with other quality parameters.

**Figure 4:**
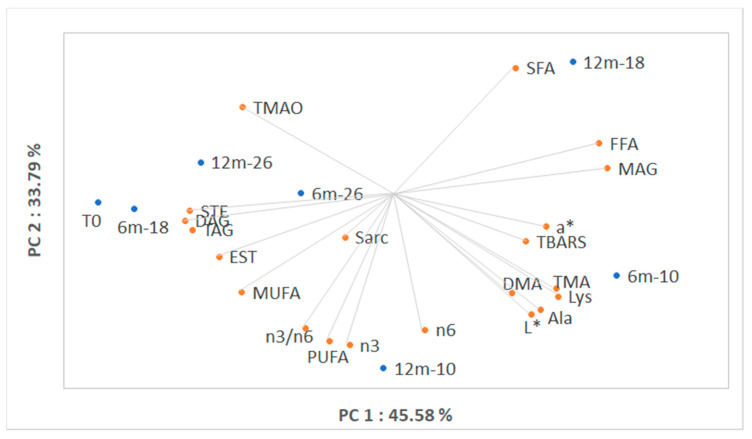
Scores and loadings biplot of data obtained from samples at the beginning of the storage (0), after 6 months (6 m) and 12 months (12 m) of storage at −10, −18 and −26 °C. PC, Principal Component; a*, red index; Ala, alanine; DAG, diacylglycerols; Dimethylamine, DMA; EST, esterified sterols; FFA, free fatty acids; L*, luminosity; Lys, lysine; MAG, monoacylglycerols; MUFA, monounsaturated fatty acids; PUFA, polyunsaturated fatty acids; Sar, sarcosine; SFA, saturated fatty acids; TAG, triacylglycerols; STE, sterols; TBARs, thiobarbituric acid reactive substances; TMA, trimethylamine-N; TMA-O, trimethylamine-O.

PCA was developed considering all parameters evaluated in this study and the score plot is reported in Figure 4. Table S3 reports the contribution of the variables to each component. Along PC1 (45.58%), samples stored at −10 °C after 6 and 12 months are clearly separated from the rest. Both samples stored at −26 °C and sample stored for 6 months at −18 °C were very close to the initial sample (0), while after 12 months of storage at −18 °C a separation occurred along the PC2 (33.79%), confirming the faster degradation rate due to increased storage temperature.

The loading plot of the variables shows that the discrimination is related mainly to the transformation of TMA-O in TMA and DMA and to lipolysis leading to the release of FFA and MAG from TAG. The different FA classes were separated mainly along PC2. The colorimetric parameter of lightness (L*) showed a higher influence on the PC1 compared to the red parameter (a*). Among the considered amino acids, alanine and lysine concentration were highly correlated to the quality degradation, while sarcosine, being close to zero, showed a weak influence.

These results confirm that the quality of mechanically separated mantis flesh subjected to proper industrial and domestic frozen storage is preserved, thus representing a suitable processing and storage technology for obtaining a valuable alternative source of n-3 and n-6 PUFA and essential amino acids for the development of innovative fish-based products addressed for human consumption. Attention should be paid in particular to avoiding the abuse of storage temperature (−10 °C) as it was demonstrated that it promoted an extensive lipid and protein degradation, due to both hydrolytic and oxidative reactions which affected the overall product quality. Whenever the frozen chain is abused, degraded frozen stored mechanically separated mantis flesh could be instead utilized for non-food sectors (animal feeding, pet food, pharmaceutical, cosmetic, etc.), thus contributing in any case to increasing the sustainability and the economic value of the overall food chain.

## 4. Conclusions

Mechanical separation and freezing of mantis shrimp flesh was carried out with the aim of valorizing an underutilized fish species from the Mediterranean characterized by high seasonality. This study demonstrated that the obtained product had a high content of PUFA, PUFA n-3 and a good PUFA n-6/PUFA n-3 ratio (< 4), confirming its high nutritional value. The separation process was shown to accelerate the initial oxidation phenomena in the flesh and to mainly promote color changes.

During frozen storage, the degradation rate was proportional to storage temperature and time, with very slight or absent quality changes at the lowest temperature (−26 °C) and a fast quality degradation at the abuse storage temperature (−10 °C). The main degradation phenomena observed were related to changes in the flesh luminosity, increase in lipid hydrolysis with the release of FFA and production of TMA and DMA probably ascribable to residual enzymatic activity and changes in amino acids concentration due to proteolytic activity.

The inter-disciplinary approach of this study permitted important findings, in terms of the extent of different degradative phenomena, related to processing and storage conditions of mechanically separated mantis flesh.

The current finding may help to develop a frozen product based on the *S. mantis* flesh characterized by a high added value aimed at the valorization of this seafood product, keeping in mind the problems related to its storage. Considering the high susceptibility of this product, in order to increase its value and its shelf-life at domestic refrigeration temperatures a possible solution could be the use of natural antioxidants added to the flesh and a strict control of the temperature during processing and storage.

## Figures and Tables

**Table 1:** Physico-chemical parameters, thiobarbituric acid reactive substances (TBARs), main lipid classes and fatty acid classes of fresh mantis flesh obtained by manual (FF) and mechanical separation (MSF).

	FF	MSF
Water content (%)	85.19 ± 0.71 ^a^	86.19 ± 0.14 ^a^
pH	6.71 ± 0.03 ^a^	6.74 ± 0.01 ^a^
L*	41.33 ± 0.05 ^a^	40.93 ± 0.09 ^b^
a*	4.77 ± 0.14 ^a^	5.56 ± 0.08 ^a^
TBARs (mg MDA/kg)	0.67± 0.03 ^b^	1.72 ± 0.31 ^a^
Total lipid content (%)	3.01 ± 0.42 ^a^	2.85 ± 0.35 ^a^
FFA (% of total lipid)	12.74 ± 2.28 ^a^	9.46 ± 0.18 ^a^
MAG (% of total lipids)	5.10 ± 0.78 ^a^	6.75 ± 0.13 ^a^
DAG (% of total lipids)	29.94 ± 0.91 ^a^	31.24 ± 0.19 ^a^
TAG (% of total lipids)	13.78 ± 8.48 ^a^	17.56 ± 0.03 ^a^
EST (% of total lipids)	28.50 ± 1.32 ^a^	28.47 ± 0.09 ^a^
STE (% of total lipids)	5.42 ± 1.38 ^a^	4.97 ± 0.26 ^a^
SFA (% of total FA)	31.27 ± 3.09 ^a^	30.17 ± 0.77 ^a^
MUFA (% of total FA)	55.72 ± 3.27 ^a^	57.87 ± 0.48 ^a^
PUFA (% of total FA)	13.00 ± 0.63 ^a^	11.95 ± 0.29 ^a^
PUFA n-3 (% of total FA)	10.18 ± 0.04 ^a^	9.35 ± 0.39 ^a^
PUFA n-6 (% of total FA)	2.82 ± 0.27 ^a^	2.78 ± 0.11 ^a^
PUFA n-6/PUFA n-3	3.64 ± 0.56 ^a^	3.69 ± 0.30 ^a^

Different letters (a–b) indicate significant differences (*p* < 0.05) between values for each considered parameter. L*, Luminosity; a*, red index; DAG, diacylglycerols; EST, esterified sterols; FFA, free fatty acids; MAG, monoacylglycerols; MUFA, monounsaturated fatty acids; PUFA, polyunsaturated fatty acids; SFA, saturated fatty acids; TAG, triacylglycerols; STE, sterols; TBARs, thiobarbituric acid reactive substances.

**Table 2:** F value and relative significance of the influence of storage time (St) and temperature (T) and their interaction (St T) on color parameters (L* and a*) and lipid oxidation (TBARs) data of fresh mantis flesh obtained by manual and mechanical separation.

	Parameter		
	L*	a*	TBARs
St	267.82 ***	48.77 ***	5.91 ***
T	2933.21 ***	0.65 ns	1.64 ns
St T	160.82 ***	13.41 ***	2.01 *

* *p* < 0.05; *** *p* < 0.001; ns: not significant. TBARs, thiobarbituric acid reactive substances.

**Table 3:** Composition of lipid classes (expressed as % of total lipids) and the fatty acid classes (expressed as % of total fatty acids) of the mechanically separated mantis flesh, after 0, 6 and 12 months storage at different temperatures.

Storage Temperature	Fat Content	FFA	MAG	DAG	TAG	STE	EST
	(%)	% of Total Lipids					
	**T0**						
**-**	2.9 ± 0.4	9.5 ± 0.2 ^d^	6.8 ± 0.1 ^de^	33.5 ±0.2 ^a^	17.6 ± 0.0 ^a^	4.9 ± 0.3 ^a^	27.9 ± 0.9 ^ab^
				**6 months**			
**−10 °C**	1.7 ± 0.1	41.3 ± 0.5 ^a^	42.3 ± 0.5 ^a^	1.9 ± 0.1 ^e^	1.4 ± 0.2 ^d^	1.3 ± 0.2 ^cd^	11.9 ± 0.4 ^de^
**−18 °C**	1.9 ± 0.1	22.2 ± 1.7 ^c^	18.0 ± 1.8 ^c^	20.8 ± 1.4 ^c^	12.1 ± 0.3 ^b^	2.7 ± 0.1 ^b^	24.2 ± 1.8 ^b^
**−26 °C**	1.8 ± 0.8	12.8 ± 0.8 ^d^	9.1 ± 0.3 ^de^	26.7 ± 0.2 ^b^	18.6 ± 0.5 ^a^	4.4 ± 0.0 ^a^	28.4 ± 0.5 ^a^
				**12 months**			
**−10 °C**	2.3 ± 1.0	47.6 ± 5.1 ^a^	39.0 ± 3.8 ^a^	0.4 ± 0.1 ^e^	1.1 ± 0.7 ^d^	0.9 ± 0.2 ^d^	11.0 ± 0.6 ^e^
**−18 °C**	2.1 ± 0.2	31.7 ± 2.4 ^b^	26.1 ± 1.6 ^b^	16.2 ±0.3 ^d^	6.3 ± 1.0 ^c^	2.0 ± 0.0 ^bc^	15.2 ± 0.2 ^cd^
**−26 °C**	2.6 ± 0.3	21.5 ± 0.9 ^c^	12.0 ± 1.4 ^d^	31.4 ± 2.2 ^a^	13.6 ± 1.4 ^b^	4.4 ± 0.5 ^a^	17.1 ± 2.1 ^c^
**Factor**				**F value**			
**St**	7.04 *	223.79 ***	254.23 ***	777.70 ***	407.13 ***	167.80 ***	287.78 ***
**T**	0.16 ns	223.38 ***	246.77 ***	692.27 ***	50.82 ***	118.70 ***	96.44 ***
**St T**	0.26 ns	31.96 ***	68.18 ***	187.70 ***	98.24 ***	31.11 ***	40.01 ***
	**SFA**	**MUFA**	**PUFA**		**n-3**	**n-6**	**n-6/n3**
	**% of total fatty acids**						
	**T0**						
**-**	30.2 ± 0.8 ^b^	57.9 ± 0.5 ^a^	12.0 ± 0.3 ^a^		9.4 ± 0.4 ^ab^	2.8 ± 0.1	3.5 ± 0.3
	**6 months**						
**−10 °C**	31.8 ± 0.6 ^ab^	55.9 ± 0.2 ^ab^	12.3 ± 0.4 ^a^		9.6 ± 0. 3 ^ab^	2.7 ± 0.1	3.5 ± 0.1
**−18 °C**	30.5 ± 1.5 ^b^	57.6 ± 0.4 ^a^	12.0 ± 1.1 ^a^		9.4 ± 1.1 ^ab^	2.6 ± 0.0	3.5 ± 0.4
**−26 °C**	29.8 ± 1.0 ^b^	57.4 ± 0.9 ^a^	12.7 ± 0.1 ^a^		10.0 ± 0.0 ^ab^	2.7 ± 0.1	3.5 ± 0.1
	**12 months**						
**−10 °C**	27.5 ± 5.1 ^b^	58.2 ± 2.9 ^a^	14.4 ± 2.1 ^a^		11.2 ± 1.1 ^a^	3.2 ± 1.1	3.6 ±0.9
**−18 °C**	40.2 ± 1.2 ^a^	51.3 ± 1.9 ^b^	7.5 ± 0.7 ^b^		5.0 ±1.5 ^b^	2.5 ± 0.6	2.9 ± 0.1
**−26 °C**	30.2 ± 1.5 ^b^	58.6 ± 0.6 ^a^	11.2 ± 0.8 ^a^		8.6 ± 0.7 ^ab^	2.6 ± 0.1	3.4 ± 0.1
**Factor**	**F value**						
**St**	2.57 ns	3.25 ns	3.29 ns		1.29 ns	0.19 ns	1.02 ns
**T**	6.95 *	5.71 *	10.59 **		5.39 *	0.57 ns	0.60 ns
**St T**	8.34 **	8.26 **	9.07 **		4.78 *	0.52 ns	0.72 ns

Different letters indicate significant differences (*p* < 0.05) among samples for each considered index. * *p* < 0.05; ** *p* < 0.01; *** *p* < 0.001; ns: not significant. DAG, diacylglycerols; EST, esterified sterols; FFA, free fatty acids; MAG, monoacylglycerols; TAG, triacylglycerols; STE, sterols; MUFA, monounsaturated fatty acids; PUFA, polyunsaturated fatty acids; SFA, saturated fatty acids.

**Table 4:** F (Fisher) values and relative significance of the influence of storage time (St) and temperature (T) and their interaction (St T) on quality parameters of mechanically separated mantis flesh, evaluated by ^1^HR-NMR.

Factor	Parameter					
	TMA-O	TMA	DMA	Lys	Sarc	Ala
St	32.58 ***	21.24 ***	16.61 ***	8.02 ***	81.18 ***	7.84 ***
T	58.10 ***	41.77 ***	68.93 ***	35.67 ***	0.35 ns	30.04 ***
St T	45.17 ***	6.30 ***	7.66 **	4.15 ***	2.17 *	3.27 **

* *p* < 0.05; ** *p* < 0.01; *** *p* < 0.001; ns: not significant. Ala, alanine; Dimethylamine, DMA; Lys, lysine; Sar, sarcosine; TMA, trimethylamine-N; TMA-O, trimethylamine-O.

**Table 5:** Correlation matrix among measured quality parameters of mechanically separated mantis flesh, after storage at different temperatures.

	L*	a*	TBARs	TMA-O	TMA	DMA	Lys	Sarc	Ala	FFA	MAG	TAG	SFA	MUFA	PUFA	n-6	n-3	n-6/n-3
L*	-																	
a*	0.570	-																
TBARs	0.530	0.429	-															
TMAO	−0.858 *	−0.478	−0.803 *	-														
TMA	0.921 *	0.377	0.561	−0.873 *	-													
DMA	0.846 *	0.237	0.158	−0.642	0.903 *	-												
Lys	0.939 *	0.462	0.647	−0.890 *	0.986 *	0.847 *	-											
Sarc	−0.099	−0.096	0.384	0.083	−0.121	−0.323	0.009	-										
Ala	0.971 *	0.456	0.610	−0.888 *	0.976 *	0.863 *	0.990 *	−0.004	-									
FFA	0.882 *	0.367	0.458	−0.874 *	0.958 *	0.909 *	0.914 *	−0.367	0.911 *	-								
MAG	0.894 *	0.412	0.638	−0.928 *	0.984 *	0.845 *	0.972 *	−0.160	0.953 *	0.969 *	-							
TAG	−0.858 *	−0.335	−0.545	0.894 *	−0.966 *	−0.864 *	−0.924 *	0.312	−0.909 *	−0.984 *	−0.981 *	-						
SFA	−0.228	0.020	0.067	0.037	0.038	−0.074	−0.023	−0.331	−0.136	0.061	0.091	−0.167	-					
MUFA	0.021	−0.139	−0.167	0.110	−0.255	−0.136	−0.206	0.266	−0.087	−0.241	−0.290	0.339	−0.963 *	-				
PUFA	0.443	0.128	0.096	−0.221	0.211	0.282	0.286	0.408	0.381	0.143	0.147	−0.041	−0.951 *	0.832 *	-			
n-6	0.513	0.155	0.088	−0.250	0.310	0.399	0.380	0.392	0.465	0.229	0.233	−0.125	−0.896 *	0.742	0.986 *	-		
n-3	0.715	0.322	−0.067	−0.344	0.574	0.767 *	0.583	−0.047	0.649	0.551	0.480	−0.430	−0.592	0.387	0.747	0.829 *	-	
n-6/n-3	0.286	−0.043	0.167	−0.136	0.103	0.111	0.193	0.630	0.273	−0.015	0.041	0.081	−0.917 *	0.820 *	0.954 *	0.927 *	0.565	-

* indicates significant correlation between parameters (*p* < 0.05). Ala, alanine; DAG, diacylglycerols; Dimethylamine, DMA; EST, esterified sterols; FFA, free fatty acids; Lys, lysine; MAG, monoacylglycerols; MUFA, monounsaturated fatty acids; PUFA, polyunsaturated fatty acids; Sar, sarcosine; SFA, saturated fatty acids; TAG, triacylglycerols; STE, sterols; TBARs, thiobarbituric acid reactive substances; TMA, trimethylamine-N; TMA-O, trimethylamine-O.

## Supplementary Materials

The following are available online at https://www.mdpi.com/2304-8158/9/10/1485/s1: Table S1: Total fatty acid composition (expressed as % of total lipids) of mechanically separated mantis flesh (MSF) and fresh mantis flesh obtained by manual (FF), Table S2: Total fatty acid composition (expressed as % of total lipids) of mechanically separated mantis flesh, after 0, 6 and 12 months of storage at different temperatures, Table S3: Factor coordinates of the variables, based on correlations.
