# Supplementary material for: Quality Changes during Frozen Storage of Mechanical-Separated Flesh Obtained from an Underutilized Crustacean
Source: Foods. 2020 Oct 17;9(10):1485. doi: 10.3390/foods9101485 (PMC7603036; doi:10.3390/foods9101485)
Supplement: Supplementary file 1 [file foods-09-01485-s001.pdf]

SUPPLEMENTARY MATERIAL

Table S1. Total fatty acid composition (expressed as % of total fatty acids) of mantis flesh obtained by manual (FF) and mechanical separation (MSF).

	C12:0	C14:0	C14:1	C15:0	C15:1	C 16:0	C 16:1 t n-7	C 16:1 t n-9	C 16:1 n-7
					(% total fatty acids)				
MSF	0.29 ±0.04	4.43 ±0.36	1.01 ±0.04	0.37 ±0.01^{a}	0.14 ±0.02	16.42 ±0.52	0.66 ±0.12^{a}	1.10 ±0.09	14.59 ±0.42
FF	0.83 ±0.31	4.41 ±0.81	0.99 ±0.11	0.26 ±0.01^{b}	0.09 ±0.01	16.88 ±1.70	0.51 ±0.08^{b}	0.92 ±0.08	12.63 ±0.77
	C 16:1 n-9	C17:0	C17:1	C18:0	C18:1 n-9	C18:1 n-11	C 18:2	C 20:0	C 18:3 n-3
					(% total fatty acids)				
MSF	0.29 ±0.06	2.07 ±0.05	1.10 ±0.01	6.04 ±0.08	16.93 ±0.15	7.03 ±0.03	1.11 ±0.02^{b}	0.29 ±0.01	0.74 ±0.13
FF	0.16 ±0.06	1.87 ±0.22	1.29 ±0.19	6.48 ±0.56	17.07 ±2.15	7.15 ±0.57	1.41 ±0.13^{a}	0.28 ±0.07	0.62 ±0.12
	C 20:1	C 20:2	C 20:3	C 22:0	C 20:5 n-3	C 22:4	C 24:1	C 22:5	C 22:6 n-3
					(% total fatty acids)				
MSF	0.41 ±0.00	0.64 ±0.06	0.07±0.00^{b}	0.27 ±0.00	5.26 ±0.36	0.86 ±0.18	14.63 ±0.54	2.39 ±0.14	0.88 ±0.02
FF	0.45 ±0.08	0.66 ±0.09	0.13±0.02^{a}	0.26 ±0.12	5.45 ±0.16	0.75 ±0.22	14.46 ±1.13	3.41 ±1.01	0.57 ±0.18

The results were reported as mean ± standard deviation. Different letters indicate significant differences (p<0.05) among samples for each considered index

Table S2. Total fatty acid composition (expressed as % of total fatty acids) of mechanically separated mantis flesh, after 0, 6 and 12 months of storage at different temperatures.

	**C12:0**	**C14:0**	**C14:1**	**C15:0**	**C15:1**	**C 16:0**	**C 16:1 t n-7**	**C 16:1 t n-9**	**C 16:1 n-7**
					(% total fatty acids)				
					**T0**				
-	0.29±0.04	4.43±0.36	1.01±0.04	0.37±0.01^{b}	0.14±0.02	16.42±0.52	0.66±0.12	1.10±0.10	14.59±0.42^{ab}
					**6 months**				
-10 °C	0.30±0.02	4.83±0.17	1.04±0.01	0.41±0.01^{b}	0.17±0.02	17.35±0.41	0.68±0.08	1.22±0.04	14.01±0.22^{b}
-18 °C	0.28±0.06	4.31±0.56	0.98±0.08	0.38±0.01^{b}	0.14±0.01	16.65±1.17	0.71±0.14	1.09±0.06	14.61±0.87^{ab}
-26 °C	0.26±0.02	4.28±0.39	0.98±0.05	0.37±0.00^{b}	0.14±0.01	16.21±0.58	0.66±0.02	1.17±0.04	14.17±0.34^{ab}
					**12 months**				
-10 °C	0.27±0.01	4.31±0.22	0.90±0.07	0.40±0.00^{b}	0.17±0.04	14.59±3.14	0.60±0.09	0.97±0.04	13.69±0.06^{b}

-18 °C	0.36±0.06	4.12±0.56	1.57±0.05	0.54±0.01^{a}	0.19±0.01	21.67±1.35	0.78±0.11	1.00±0.06	19.51±0.79^{a}
-26 °C	0.30±0.07	4.47±0.75	1.01±0.08	0.39±0.03^{b}	0.16±0.02	16.33±0.75	0.76±0.06	1.07±0.04	14.78±0.59^{ab}
Factor					**F value**				
St	0.59 ns	1.22 ns	31.34****	23.92 ***	5.09 *	0.05 ns	0.12 ns	8.47 **	0.2.36 ns
T	0.59 ns	1.21 ns	62.03 ***	16.4 ***	0.964 ns	0.44 ns	0.37 ns	1.45 ns	4.38 *
St T	0.66 ns	1.75 ns	8.17 ***	18.07 ***	0.92 ns	0.76 ns	0.51 ns	1.08 ns	2.92 ns

	**C 16:1 n-9**	**C17:0**	**C17:1**	**C18:0**	**C18:1 n-9**	**C18:1 n-11**	**C 18:2**	**C 20:0**	**C 18:3 n-3**
					(% total fatty acids)				
					**T0**				
-	0.29±0.06	2.07±0.05	1.10±0.01	6.04±0.08	16.93 ±0.15^{ab}	7.03±0.03	1.11±0.02	0.29±0.01	0.74±0.13
					**6 months**				
-10 °C	0.40±0.00	2.00±0.00	1.03±0.06	6.30±0.00	16.85±0.03^{ab}	6.64±0.17^{ab}	1.06±0.02	0.31±0.00	0.83±0.02
-18 °C	0.30±0.09	2.11±0.02	1.12±0.01	6.16±0.20	17.08±0.01^{ab}	6.89 ±0.04^{a}	1.12±0.02	0.29±0.04	0.88±0.05
-26 °C	0.25±0.01	1.99±0.06	1.09±0.02	6.13±0.05	16.97±0.40^{ab}	6.94±0.30^{a}	1.08±0.00	0.30±0.02	0.87±0.01
					**12 months**				
-10 °C	0.66±0.306	1.88±0.22	1.14±0.14	5.41±1.48	18.28±1.32^{a}	7.19±0.46^{a}	1.67±0.90	0.30±0.03	0.84±0.02
-18 °C	0.33±0.088	2.34±0.02	1.34±0.01	5.48±0.18	11.42±0.70^{b}	5.83±0.04^{b}	1.08±0.01	0.20±0.03	0.49±0.04
-26 °C	0.34±0.032	2.13±0.02	1.41±0.20	6.04±0.13	17.46±0.54^{a}	6.92±0.086^{a}	1.11±0.01	0.29±0.02	0.60±0.02
Factor					**F value**				
St	3.70 ns	0.18 ns	3.32 ns	2.47 ns	0.82 ns	4.93 *	1.32 ns	3.77 ns	8.27 **
T	3.27 ns	1.92 ns	1.39 ns	0.37 ns	4.74 *	6.13 *	0.46 ns	6.14 *	0.25 ns
St T	1.28 ns	1.17 ns	1.06 ns	0.51 ns	5.33 *	9.02 **	0.57 ns	3.49 ns	0.09 ns

	**C 20:1**	**C 20:2**	**C 20:3**	**C 22:0**	**C 20:5 n-3**	**C 22:4**	**C 24:1**	**C 22:5**	**C 22:6 n-3**
					(% total fatty acids)				
					**T0**				
-	0.41±0.00^{ab}	0.64±0.06	0.07±0.00	0.27±0.00	5.26±0.36	0.86±0.18	14.63±0.54^{a}	2.39±0.14^{b}	0.88 ±0.02^{ab}
					**6 months**				
-10 °C	0.46±0.00^{ab}	0.63±0.00	0.09±0.00	0.28±0.03	4.94±0.06	0.98±0.02	13.40±0.28^{a}	2.89 ±0.20^{ab}	0.87 ±0.02^{ab}
-18 °C	0.41±0.03^{ab}	0.63±0.07	0.07±0.00	0.28±0.04	5.24±0.51	0.83±0.04	14.25 ±1.01^{a}	2.30 ±0.41^{b}	0.89 ±0.12^{ab}
-26 °C	0.42±0.00^{ab}	0.62±0.02	0.09±0.00	0.30±0.05	5.18±0.21	1.05±0.09	14.63±0.58^{a}	2.92 ±0.27^{ab}	0.92 ±0.07^{ab}

				**12 months**					
-10 °C	0.60 ±0.15^{a}	0.68±0.08	0.09±0.00	0.32±0.01	5.18±0.43	0.81±0.09	13.98±1.50^{ab}	4.13 ±0.57^{a}	0.96 ±0.08^{a}
-18 °C	0.27±0.03^{b}	0.43±0.07	0.07±0.00	0.17±0.03	3.72±0.51	0.60±0.04	10.40±1.00^{b}	1.21 ±0.42^{b}	0.41 ±0.11^{b}
-26 °C	0.40 ±0.00^{ab}	0.63±0.02	0.08±0.00	0.28±0.02	5.23±0.32	0.83±0.13	14.26±0.49^{a}	1.80± 2.30^{b}	0.91 ±0.08^{ab}
Factor				**F value**					
St	0.527 ns	0.33 ns	3.75 ns	0.72 ns	1.02 ns	3.40 ns	7.57 *	1.26 ns	1.41 ns
T	4.54 *	1.08 ns	0.69 ns	1.96 ns	0.68 ns	1.74 ns	4.68 *	14.41 **	3.84 ns
St T	2.73 ns	1.17 ns	1.06 ns	1.91 ns	1.29 ns	0.45 ns	5.34 *	10.14 **	3.77 *

The results were reported as mean ± standard deviation. Different letters indicate significant differences (p<0.05) among samples for each considered index. * p<0.05; **p<0.01; ***p<0.001; ns: not significant

Table S3. Factor coordinates of the variables, based on correlations

	Factor 1	Factor 2	Factor 3	Factor 4	Factor 5	Factor 6
L*	0.6108	-0.7458	-0.1630	-0.0045	0.1380	-0.1583
a*	0.6760	-0.2007	0.3408	0.5374	0.2140	-0.2280
TBARS	0.5876	-0.2926	0.5147	-0.4770	0.2763	0.0216
FFA	0.9170	0.3149	0.1142	0.1913	-0.0506	0.0876
MAG	0.9535	0.1595	0.2337	0.0517	-0.0806	0.0410
EST	-0.7984	-0.3857	-0.2256	-0.3345	-0.2158	-0.0671
DAG	-0.9540	-0.1628	-0.1154	0.0687	0.1911	-0.0937
STE	-0.9312	-0.1048	-0.0975	-0.1754	0.2706	-0.0916
TAG	-0.9193	-0.2219	-0.2238	-0.2262	0.0656	0.0065
SFA	0.5392	0.7810	-0.1773	-0.2198	-0.1196	-0.0721
MUFA	-0.6969	-0.6062	0.1764	0.2081	0.2588	0.0743
PUFA	-0.3015	-0.9079	0.2073	0.1897	-0.0353	0.0677
n3	-0.2095	-0.9326	0.1429	0.1986	-0.1530	0.0559
n6	0.1315	-0.8387	-0.2706	0.3859	-0.2389	-0.0010
n3/n6	-0.4081	-0.8309	0.3529	-0.0265	-0.1147	0.0682
TMAO	-0.6929	0.5372	-0.0001	0.2803	-0.3721	-0.1195
TMA	0.7224	-0.5852	-0.2769	-0.2415	-0.0253	-0.0048
DMA	0.5237	-0.6116	-0.5734	-0.0156	-0.1492	0.0172
Lys	0.7319	-0.6322	-0.1190	-0.2191	-0.0386	-0.0310

Sarc	-0.2297	-0.2679	0.7530	-0.3538	-0.4111	-0.1195
Ala	0.6538	-0.7149	-0.1475	-0.1852	0.0204	-0.0702
